# A Halogen Bonding Perspective on Iodothyronine Deiodinase Activity

**DOI:** 10.3390/molecules25061328

**Published:** 2020-03-14

**Authors:** Eric S. Marsan, Craig A. Bayse

**Affiliations:** Department of Chemistry and Biochemistry, Old Dominion University, Norfolk, VA 23529, USA; emarsan@odu.edu

**Keywords:** iodothyronine deiodinase, halogen bonding, xenobiotics, endocrine disruption, polybrominated diphenyl ethers (PBDEs), polychlorinated biphenyls (PCBs), thyroid hormones (THs)

## Abstract

Iodothyronine deiodinases (Dios) are involved in the regioselective removal of iodine from thyroid hormones (THs). Deiodination is essential to maintain TH homeostasis, and disruption can have detrimental effects. Halogen bonding (XB) to the selenium of the selenocysteine (Sec) residue in the Dio active site has been proposed to contribute to the mechanism for iodine removal. Polybrominated diphenyl ethers (PBDEs) and polychlorinated biphenyls (PCBs) are known disruptors of various pathways of the endocrine system. Experimental evidence shows PBDEs and their hydroxylated metabolites (OH-BDEs) can inhibit Dio, while data regarding PCB inhibition are limited. These xenobiotics could inhibit Dio activity by competitively binding to the active site Sec through XB to prevent deiodination. XB interactions calculated using density functional theory (DFT) of THs, PBDEs, and PCBs to a methyl selenolate (MeSe^−^) arrange XB strengths in the order THs > PBDEs > PCBs in agreement with known XB trends. THs have the lowest energy C–X*-type unoccupied orbitals and overlap with the Se lp donor leads to high donor-acceptor energies and the greatest activation of the C–X bond. The higher energy C–Br* and C–Cl* orbitals similarly result in weaker donor-acceptor complexes and less activation of the C–X bond. Comparison of the I···Se interactions for the TH group suggest that a threshold XB strength may be required for dehalogenation. Only highly brominated PBDEs have binding energies in the same range as THs, suggesting that these compounds may inhibit Dio and undergo debromination. While these small models provide insight on the I···Se XB interaction itself, interactions with other active site residues are governed by regioselective preferences observed in Dios.

## 1. Introduction

Thyroid hormones (THs) are essential biomolecules involved in many biochemical processes, particularly in early developmental stages [1,2,3,4,5]. The prohormone thyroxine (3,3′,5,5′-tetraiodothyronine, T_4_), and to a lesser extent, 3,3′,5-triiodothyronine (T_3_) are secreted from the thyroid gland upon stimulation by thyroid stimulating hormone (TSH) [6]. Transport proteins (TPs), such as thyroglobulin (TBG) and transthyretin (TTR), transport THs to target cells based on metabolic and/or developmental needs [1].

**Figure 1:**
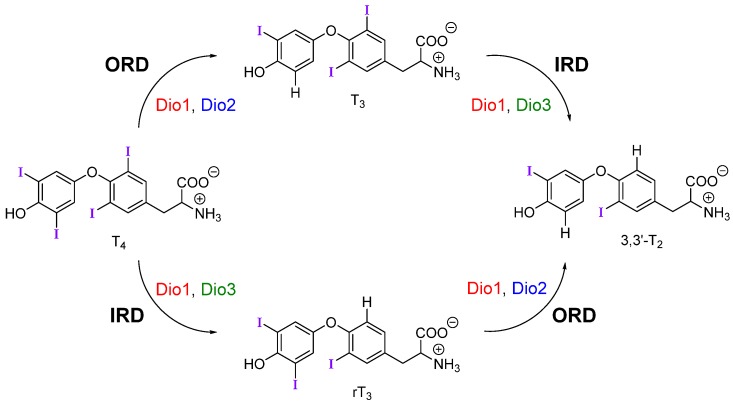
Mechanistic pathways of deiodination by each deiodinase with thyroid hormone (TH) substrates. Dio is regioselective for outer-ring or inner-ring deiodination (ORD and IRD, respectively).

Upon reaching target cells, deiodination by the iodothyronine deiodinase (Dio) family of selenoproteins modulates TH signaling by controlling levels of the active metabolite T_3_ (Figure 1) [1]. Deiodination of the outer (phenolic) ring or inner (tyrosyl) ring of THs are activating and inactivating pathways respectively. For example, outer-ring deiodination (ORD) of T_4_ by Type I (Dio1) or Type II (Dio2) deiodinases produces active T_3_, while inner-ring deiodination (IRD) of T_4_ by Type III (Dio3, and Dio1 to a lesser extent) produces the inactive metabolite 3,3′,5′-triiodothyronine or reverse T_3_ (rT_3_) (Figure 1). Dio3 also lowers T_3_ concentrations through conversion to 3,3′-diiodothyronine (T_2_). Deiodination is facilitated by a rare selenocysteine (Sec) residue within the cleft of the active site [7].

Disruption of TH homeostasis by xenobiotics can have long-term negative health effects such as structural abnormalities, cardiovascular diseases, and hypo/hyperthyroidism [1,8]. Organohalogen compounds, such as polybrominated diphenyl ethers (PBDEs) and polychlorinated biphenyls (PCBs), are known endocrine-disrupting compounds that induce a range of developmental and neurodegenerative effects [9,10,11,12,13,14,15,16,17,18,19,20]. Recently, studies have shown that inhibition of Dio activity may be one pathway for disruption [21,22,23,24]. Related halogenated compounds such as polybrominated biphenyls (PBBs) and polychlorinated diphenyl ethers (PCDEs) have been shown to alter TH levels but have not yet been shown to inhibit Dio [25,26,27].

**Figure 2:**
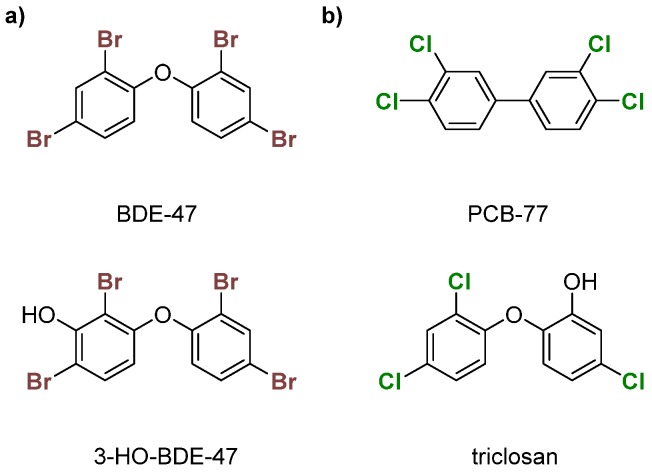
Examples of (**a**) polybrominated diphenyl ethers (PBDEs)—BDE-47 and 3-HO-BDE-47; (**b**) polychlorinated biphenyls (PCBs)—PCB-77 and triclosan.

PBDEs are used in commercial products to increase flame resistance (Figure 2a) [28,29]. However, PBDEs contaminate house dust, leading to exposure via ingestion or inhalation [21]. As a result, some formulations, namely the penta- and octa-BDEs, were banned in the early 2000s [30,31]. Industrial runoff of these compounds into the environment has led to bioaccumulation in organisms over time, leading to contamination in wildlife [32,33,34,35,36]. Enzymatic debromination of higher-order PBDEs (>5 Br) contributes to more efficient bioaccumulation [30,37]. Hydroxylated metabolites (OH-BDEs) have been shown to inhibit TRβ in silico and in vitro [38,39]. There is evidence for Dio inhibition by PBDEs and OH-BDEs in fish, birds, and humans [21,37,40,41].

PCBs, like PBDEs, are industrial flame retardants with high chemical stability (Figure 2b) [42,43]. Production of some PCB formulations were banned in the 1970s, but they still contaminate urban areas [44,45,46,47]. These organohalogens are classified into two subcategories—coplanar or dioxin-like (having no ortho chlorines) and non-coplanar or non-dioxin-like (having one or more ortho chlorines). Dioxin-like PCBs are highly toxic, which is often attributed to an assumed structural similarity with tetrachlorodibenzodioxin (TCDD), a known inhibitor of the aryl hydrocarbon receptor (AhR) [48]. Non-dioxin-like PCBs are toxic at higher concentrations and inhibit TBG and TTR [49,50,51,52]. PCBs have been reported to disrupt TH homeostasis through other mechanisms, such as the sodium-iodide symporter (NIS) [51,53,54,55,56]. Limited experimental data suggest that PCBs disrupt TH levels, which could indicate Dio inhibition [57,58,59]. The hydroxylated compound triclosan has been shown to weakly inhibit Dio (Figure 2b) [60].

**Figure 3:**
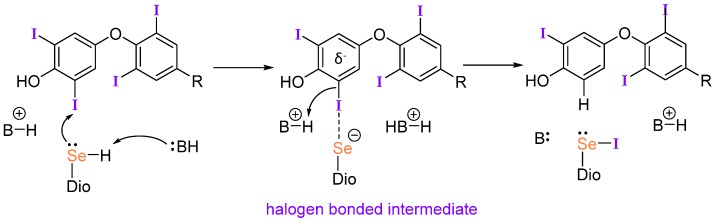
Proposed halogen bonding-based mechanism for deiodination by Dio adapted from reference [71]. The identities of B and their protonation states have not been determined.

Halogen bonding (XB) has gained importance in drug design and crystal engineering [61,62,63,64,65,66,67,68,69,70]. Our group has proposed that XB participates in the Dio mechanism through the formation of an initial Se···I interaction between the selenium of the active site Sec residue and a TH iodine (Figure 3) [71]. This mechanism is supported by the work of Mugesh et al. on naphthyl-based deiodinase mimics which display high activity through a combination of halogen and chalcogen bonding [72]. In addition, Schweizer et al. identified potential proton channels in their X-ray structure of the Dio3 catalytic domain that support the XB-based mechanism [7]. Dios prefer the rare Sec residue due to its high nucleophilicity relative to Cys, which is enhanced by deprotonation at physiological conditions. Recent studies by our group explored the possibility that organohalogens like PCBs and PBDEs could inhibit Dio activity by blocking the active site through an X···Se halogen bonding (XB) interaction [28,73].

**Figure 4:**
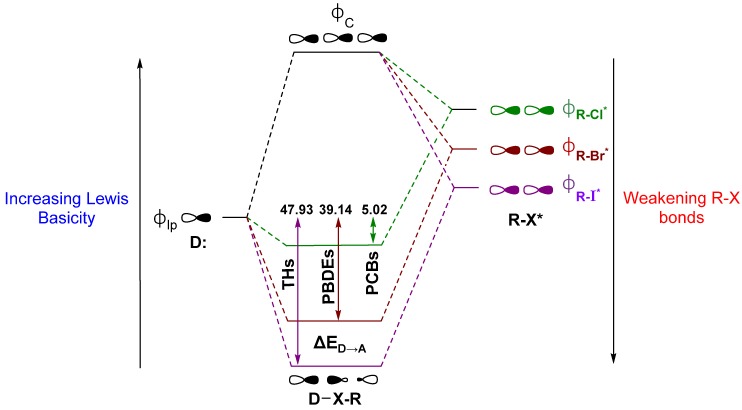
XB as described by the molecular orbital (MO) model showing the interaction between a lone pair of a donor and the R–X antibonding orbital, alongside the corresponding average stabilization of the Se donor lone pair by THs, PBDEs, and PCBs as determined by Natural Bond Orbital (NBO) ΔE_D→A_ analysis. Units are kcal mol^−1^. Adapted from reference [66].

An ongoing debate on the driving forces for the XB interaction has raged in the literature [42,66,74,75,76,77,78,79,80,81,82]. Briefly stated, one side describes XB as driven primarily by electrostatics, where the donor interacts with an area of positive electrostatic potential on the distal end of the R–X bond, commonly called the “σ-hole” [68,83,84]. This “polar flattening” results from electron density depletion along the R–X bond axis, leading to the halogen to adopt an anisotropic, oblate shape [67,85,86,87]. Groups on the other side note charge transfer as a significant contributor to XB and use descriptions in terms of valence bond theory or molecular orbital (MO) interactions related to early contributions by Mulliken [71,80,88,89,90,91,92,93,94]. Our group’s discussion of XB in Dio activity has focused on this latter MO description to define XB as a donor-acceptor interaction between the lone pair of a nucleophile (σ_lp_) and the antibonding orbital (σ_R-X_*) on the acceptor fragment (Figure 4). According to this model, XB is strengthened for (a) Lewis acids with weaker R–X bonds, which have lower lying σ_R-X_* MOs, and (b) stronger Lewis bases due to destabilization of the lp donor MOs. In Dio, a partial explanation for the preference of Sec of Cys is the greater Lewis basicity of the selenolate over the thiolate [71]. In peri-chalcogen-substituted naphthyl-based Dio mimics, strong nucleophiles have higher Dio-like activity (i.e., Se,Se > Se,S > S,S), consistent with the preference for selenium over sulfur [95,96]. Natural Bond Orbital (NBO) theory can be used to calculate the donor-acceptor energy (ΔE_D→A_) as the extent to which donation into the σ_R-X_* acceptor stabilizes of the lp donor MO (Figure 4) [71,97]. The trend in ΔE_D→A_ for organohalogens is consistent with increasing XB strength with halogen size. The increased donation into σ_R-X_* leads to more activated C–X bonds (i.e., C–I > C–Br > C–Cl for both Δ*d*(C–X) and ΔE_D→A_) (Figure 4) [28].

Overlap with the donor MO is enhanced when the acceptor σ_R-X_* MO has a greater contribution from the halide AOs. Decreasing the electronegativity from F to I causes the X AOs to destabilize relative to the R fragment, resulting in orbitals with more ‘R’-like character in σ_R-X_ while increasing the X contribution (%X) to σ_R-X_* [66]. The shift in the R character of σ_R-X_ depletes the electron density along the bond axis only, consistent with the observation of a σ-hole in the electrostatic potential [84]. XB interactions have also been described using a more complex MO diagram for the mixing of both σ_R-X_ and σ_R-X_* with the donor MO, resulting in an interaction similar to the 3c4e hypervalent bond observed in the trihalide I_3_^−^ (Figure 4) [89,90,98,99,100]. Maximization of the overlap between σ_lp_ and σ_R-X_* requires a near 180° R–X···Y angle, where Y is the donor. Many protein-ligand XB interactions fall in the range of 140° to 160° [65,101,102,103].

## 2. Summary of XB Models Related to Dio Activity

### 2.1. Thyroid Hormone (TH) I···Se XB Interactions

XB is observed in X-ray structures of TH binding proteins, such as TTR and TBG, primarily in the form of weak I···O bonds (i.e., PDB entries 1SNO, 2CEO) [71,104,105,106]. I···Se XB interactions are found in the T_4_ lysozyme (selenomethionine mutant, PDB 3DN3) as well as aromatic crystalline systems [91,107]. In Dio, the nucleophilic selenium is assumed to form a strong I···Se XB interaction which activates the C–I bond (Figure 3). Trends in density functional theory (DFT) interaction energies for the series of THs (T_4_, T_3_, rT_3_, 3,3′-T_2_, 3,5-T_2_, 3′,5′-T_2_, 3-T_1_, 3′-T_1_) were calculated using MeSe^−^ as a minimalistic model of Dio’s active site Sec at each unique position on the outer and inner rings [28]. The stronger nucleophilicity of MeSe^−^ results in larger donor-acceptor energy interactions compared to sulfur analogues [71,72]. Manna et al. found the same trends in XB interactions of T_4_,T_3,_ rT_3_, and 3,3′-T_2_ using methylselenol (MeSeH), but with interactions approximately an order of magnitude weaker due to the neutral donor [72]. In either case, trends in XB strength do not correlate with Dio regioselectivity, which is governed by other factors, such as interactions with residues within the active site that are omitted from the simple DFT models. For example, weaker I···O and I···N XB interactions between ancillary iodines and adjacent residues may help stabilize the TH substrate within the active site [104,108]. These additional XB interactions may enhance the preference towards the outer or inner ring and stabilize the substrate within the active site. The conformation of the TH may also be important, as substrates may need to fit in the active site in a certain way to allow for binding. Crystallographic data from the Protein Data Bank suggest proteins bind THs in cisoid (both phenyl rings on same side as ether linkage) or transoid (both phenyl rings on opposite sides of ether linkage) conformations [104].

**Figure 5:**
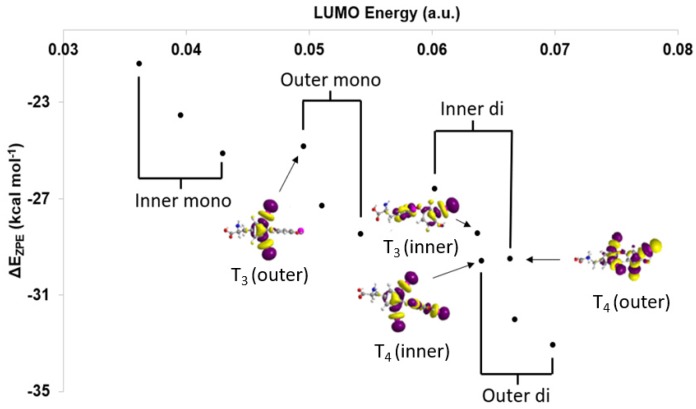
Comparison of lowest unoccupied molecular orbital (LUMO) energies with respect to ΔE_ZPE_ and select LUMOs of the inner and outer rings of T_4_ and T_3_. Adapted from reference [28].

Based upon the MO model (Figure 4), XB trends are understood in terms of the orbital energy and C–I* character of the lowest unoccupied molecular orbitals (LUMOs) [28]. In addition, XB favorability and activation of the C–X bond can be calculated in terms of corrected zero-point interaction energies (ΔE_ZPE_) for formation of the XB complex and NBO donor-acceptor energies (ΔE_D→A_), respectively [28,71]. XB is generally more favorable for diiodinated rings compared to monoiodinated rings (Table 1). Increased donation into the σ_R-I_* MO leads to stronger I···Se interactions and a larger activation of the C–I bond in agreement with the MO model. Interaction strengths positively correlate with C–I* LUMO energies within each TH subgroup (inner-mono < outer-mono ≤ outer-di < inner-di) (Figure 5) [28]. These results suggest that specific substitution patterns, such as outer-diiodinated THs, may be more suitable for targeting Dio2, while inner-diiodinated THs prefer Dio3. This prediction is consistent with the preferred substrates of Dio2 (rT_3_) and Dio3 T_3_ (3,5-diiodothyronine) [109]. For example, based upon DFT XB strengths, the deiodination reaction of T_4_ would first occur at the outer-ring (T_3_) with subsequent deiodination on the opposite ring to 3,3′-T_2_ (Table 1 and Figure 2) [28]. These results are consistent with the preferred substrate and regioselectivity of Dio3, which favors T_3_ (IRD). However, these results do not represent a general trend in Dio regioselectivity given that Dio2 acts upon the outer ring iodine of T_4_, which has a weaker interaction than the inner ring. Additionally, 3-T_1_ is not deiodinated to thyronine (T_0_) and has the least favorable XB interaction to SeMe^−^, suggesting that a threshold interaction strength must be met to cleave the C–I bond (Figure 3). For example, T_4_ has a high ΔE_ZPE_ (−29.59 kcal mol^−1^) and a more lengthened C–I bond (Δ*d*(C–I) = +0.198 Å) compared to 3-T_1_ (ΔE_ZPE_ = −21.41, Δ*d*(C–I) = +0.158 Å) (Table 1). A minimum donation into σ_R-X_*, which is related to the overall strength of the R–X bond, may be required to convert the XB interaction to a nucleophilic attack. The inability of 3-T_1_ to undergo dehalogenation by Dio suggest that it may only reversibly bind to the active site of Dio [110].

### 2.2. Polybrominated Diphenyl Ether (PBDE) Br···Se XB Interactions

**Figure 6:**
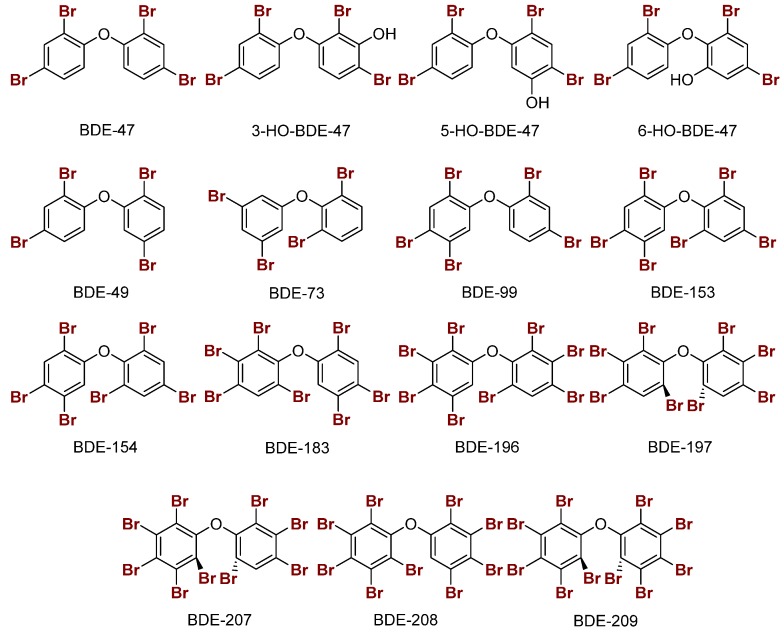
Sample structures of PBDEs and the hydroxylated metabolites of BDE-47.

Br···Se XB interactions were modeled for a selection of PBDEs and hydroxylated PBDEs (OH-BDEs) with MeSe^−^ at each unique halogen position (ortho, meta, or para) (Figure 6) [21]. In general, XB interactions of PBDEs are less favorable compared to THs, consistent with the XB trends favoring larger halogens (I > Br > Cl). In PBDEs, XB interactions are favored at the ortho and meta positions which have more activated C–Br bonds. Increased halogenation of PBDEs generally leads to increased activation of C–Br bonds due to lowering of the σ_R-X_* acceptor MOs for stronger interactions. XB favorability in PBDEs/OH-BDEs is consistent with the observed ortho and meta iodination positions of THs. The stronger XB interactions at the ortho and meta positions of halogenated diphenyl ethers may be related to the adaptation of THs to biological systems in conjunction with the higher nucleophilicity of Sec. PBDEs with ortho and meta substitutions, such as BDE-73, may be better inhibitors of Dio [28].

**Figure 7:**
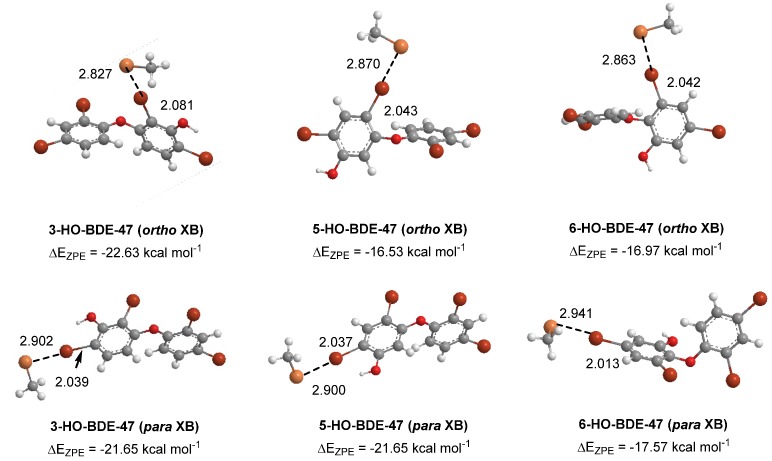
Density functional theory (DFT) optimized structures of XB complexes at the ortho and para positions of hydroxylated BDE-47 metabolites. Distances are in Angstroms.

**Figure 8:**
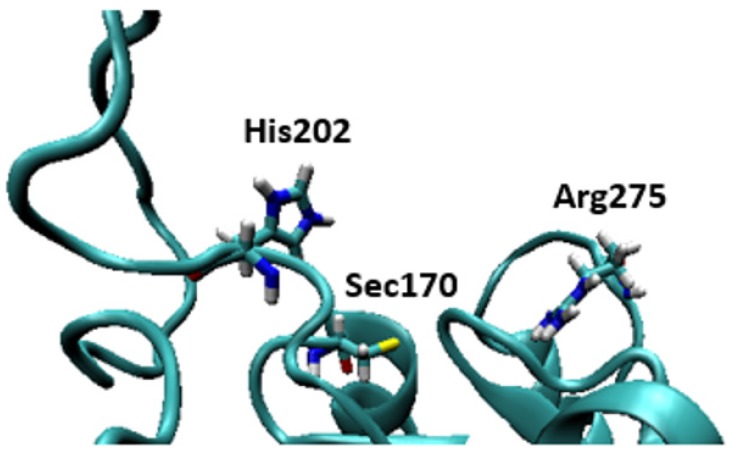
Structures of the active site of the crystal structure of Dio3 (PDB = 4TR4) with Sec170 and the residues of the His202-Arg275 clamp proposed by Schweizer et al. indicated [7].

Some of the highly brominated PBDEs/OH-BDE (≥5 Br) complexes have donor-acceptor interaction energies in the same range as THs. For example, in 6-HO-BDE-47, the strong interaction at the 2′ position (ΔE_D→A_ = 44.77 kcal mol^−1^, Δ*d*(C–Br) = +0.206 Å) is in the same range of THs and could be susceptible to debromination (Figure 6). However, interaction at the 4′ position is much weaker (ΔE_D→A_ = 21.12 kcal mol^−1^, Δ*d*(C–Br) = +0.102 Å) and may be less likely to undergo debromination (Figure 6). In DFT studies, XB favorability was enhanced by the proximity of -OH to the XB interaction site [28]. For example, in BDE-47, XB is more favorable at the ortho (ΔE_ZPE_ = −17.54 kcal mol^−1^) compared to para (ΔE_ZPE_ = −16.62 kcal mol^−1^). However, upon hydroxylation, XB at the para position of 5-HO-BDE-47 (ΔE_ZPE_ = −21.65 kcal mol^−1^) is enhanced by the proximal OH group for a more favorable interaction than ortho (ΔE_ZPE_ = −16.53 kcal mol^−1^) (Figure 7). In OH-BDEs, the -OH group may aid in substrate recognition to Dio by better mimicking TH binding. Schweizer et al. proposed that T_4_ was held in the Dio3 active site by a His202-Arg275 clamp through which His202 forms a hydrogen bond to the T_4_ 4′-OH group (Figure 8) [7].

Various studies suggest the potential for PBDEs to inhibit Dio to varying degrees [21,60,111]. For example, François et al. found an increase in Dio1 activity at a 1.0 nM concentration of BDE-209, but not at 0.5 nM or 2.0 nM [41]. In addition, 3-HO-BDE-47 inhibits Dio2, although 6-HO-BDE-47 showed no activity [21]. The difference in XB favorability between 3-HO-BDE-47 and 6-HO-BDE-47 suggest hydrogen bonding of a hydroxyl group adjacent to the XB-interacting Br may aid substrate binding in TH binding proteins. In comparing Dio2 inhibition activity of 3-HO-BDE-47 and 5′-HO-BDE-99, the former had a more favorable XB interaction to our small active site model (at the para position adjacent to the –OH group) [60,111]. Factors such as interactions at the active site not included in our minimalistic model may govern the preference of Dio2 inhibition. In another study, a series of pentabrominated PBDEs (BDE-28, 33, 47, and 100) (ΔE_ZPE_ ≈ −13.00 to −23.00 kcal mol^−1^) showed a positive correlation between concentration and free T_3_ levels, suggesting Dio1 inhibition. However, other mechanisms may be disrupted, such as displacement of THs from transport proteins [112].

Animal studies have shown that PBDEs are debrominated by Dios [21,41,113]. Out of a series of 20 PBDEs, Roberts et al. showed that six (BDEs 99, 153, 183, 203, 208, and 209) (ΔE_ZPE_ ≈ −19.00 to −29.00 kcal mol^−1^) undergo debromination in common carp, rainbow trout, and chinook salmon [21]. The interaction energies for these examples are close to or exceed that of 3-T_1_, providing support for the potential for a threshold XB strength needed for dehalogenation. Across all three species, meta-substituted PBDEs were the preferred substrates, however the preference for debromination varied across species. In carp, debromination was preferred at the meta position, while trout and salmon preferred debromination at the ortho position [21]. They found that neither BDE-49 nor BDE-154 undergo dehalogenation in any of the species. Avian studies have shown that consumption of BDE-209 lead to increased concentrations of octa- and nona-BDEs (BDEs 196, 197, 203, 207, and 208) [41,113]. The differences in observed debrominated products and regioselective preferences for debromination suggest different Dios are targeted across species. For instance, in carp, preference for deiodination at the meta position suggests a higher affinity for Dio2, while the ortho preference in trout and salmon may suggest better inhibition of Dio3.

### 2.3. Polychlorinated Biphenyl (PCB) Cl···Se XB Interactions

**Figure 9:**
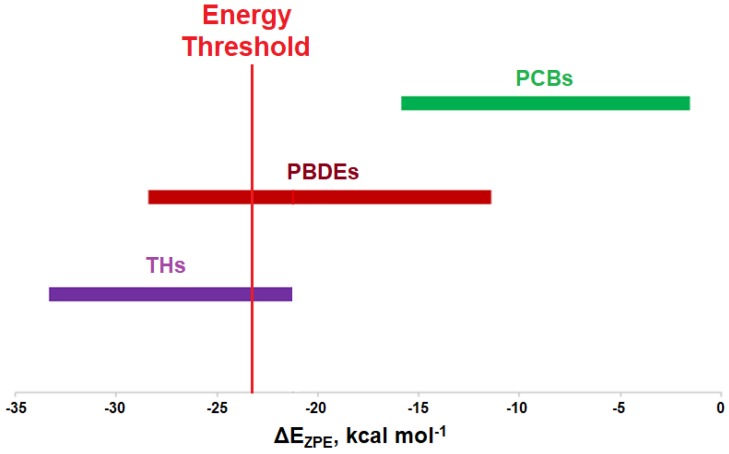
Comparison of XB interactions of THs, PBDEs, and PCBs. The red line indicates a proposed energy threshold needed for dehalogenation, based upon the interaction energy for 3-T_1_.

**Figure 10:**
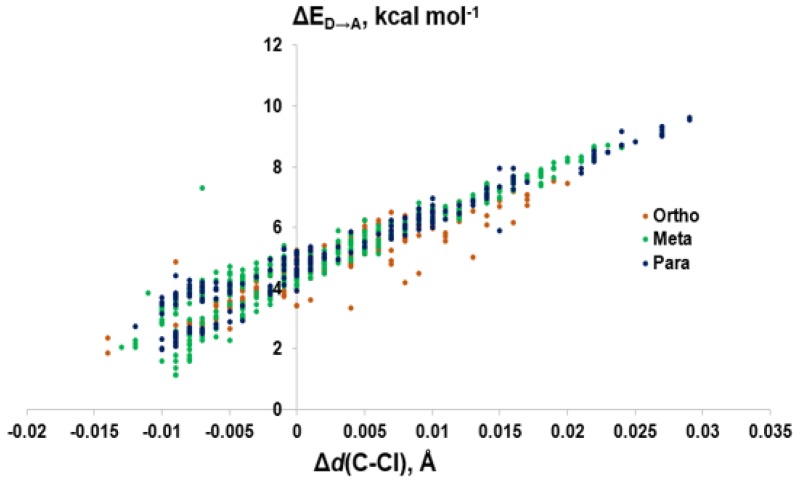
Comparison of donor-acceptor energies (ΔE_D→A_) to the activation of the C–Cl bond (Δ*d*(C–Cl)) by XB position.

**Figure 11:**
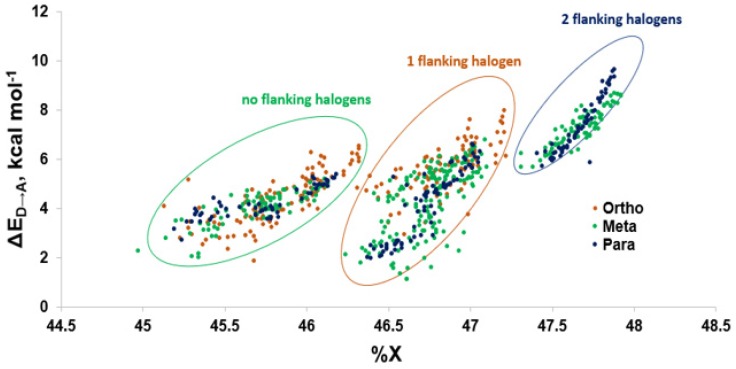
Comparison of donor-acceptor energies (ΔE_D→A_) to percent contribution of X (%X) by XB position.

Modeling of the Cl···Se XB interactions for all possible 209 PCB congeners at each unique XB position found that PCBs have much weaker XB interactions than PBDEs and THs (Figure 9), as expected based on the trend of XB strength in the order of I > Br > Cl [73]. XB interactions for PCBs are more favorable at the ortho position, consistent with PBDE XB interactions. On average, meta and para XB interactions that contain two flanking halogens cluster towards a higher ΔE_D→A_ (Figure 10 and Figure 11). None of the PCBs, even the highly chlorinated compounds, have ΔE_ZPE_ values in the range of THs, suggesting dechlorination by Dio is unlikely (Figure 8).

The weaker XB interactions in PCBs is attributed to their stronger C–Cl bonds and the higher energy σ_C-Cl*_ acceptor orbitals compared to the bromo- and iodoaromatics [66]. The C–Cl bonds are so strong that the π* MOs are the LUMOs rather than the σ_C-Cl*_-type orbitals. These orbitals are also less weighted toward Cl, leading to less overlap with the Se lp. (%X contributions to σ_C-Cl_^*^ in PCBs are 45–48% compared to aryl bromides (50–55%) and aryl iodides (55–65%) (Figure 9) [66]. Meta and para XB interactions also generally have higher %X values compared to ortho XB interactions, consistent with their stronger ΔE_D→A_ values. The slightly more favorable XB interactions for meta and para chlorides flanked by two halogens are attributed to the electron-withdrawing properties of the neighboring substituents, which stabilizes the σ_C-Cl_^*^ acceptor MO [73].

Experimental evidence for Dio inhibition by PCBs is limited and shows conflicting results [57,58,114]. For example, Dio3 activity increased in the brain upon exposure to Aroclor 1254, a mixture containing primarily PCB-77, but hepatic Dio1 and brain Dio2 activity decreased [114]. In contrast, neither PCB-77 nor Aroclor 1254 had an effect on brain Dio2 activity in adult mice [58]. A recent study involving cord blood showed a positive correlation between the concentration of 2,4,5-substituted PCBs and the T_3_/rT_3_ ratio, indicating Dio3 inhibition [57]. This negative effect on Dio3 activity is consistent with trends for highly chlorinated PCBs and a hypothesis of blocking TH binding through an XB interaction. Trends in XB favorability are also similar to a study involving TTR inhibition, which compared substitution patterns of PCBs [115].

## 3. Discussion

Modeling the XB interactions of halogenated endocrine disruptors with the Dio active site model SeMe^−^ model provide insight into potential mechanisms of inhibition. Xenobiotics such as PBDEs and PCBs may inhibit Dio by forming an X···Se XB interaction to the catalytic Sec in the active site. XB favorability in the order of THs > PBDEs > PCBs agrees with the expected trends (I > Br > Cl). THs generally undergo deiodination by Dios, an exception being 3-T_1_ which has the weakest I···Se XB interaction of the series. This observation suggests that XB interaction strength with the active site Sec must exceed an energy threshold for deiodination. While some of the highly substituted PBDEs/OH-BDEs have similar interaction energies to THs and may undergo debromination, PCBs have less favorable interactions, suggesting dechlorination by Dio would not be observed.

XB interaction strengths vary by position. The preference for XB at the meta and ortho positions of diphenyl ethers suggests that the substitution pattern of THs may have been selected to facilitate enzymatic deiodination. The position dependence of XB interactions of PBDEs (ortho and meta) and PCBs (meta and para) suggest that these compounds may target Dio types with substitution patterns similar to their preferred substrate [116]. For example, Dio1 performs both ORD (meta-) and IRD (ortho-) (although there is a preference for ORD) with rT_3_ as its preferred substrate, while Dio2 and Dio3 prefer ORD (meta-) and IRD (ortho-) with T_4_ and T_3_, respectively. A PCB, PBDE or related compound with a structure containing solely meta halogens (such as PCB-80 or BDE-80), or its strongest XB interactions at the meta position, may preferentially inhibit Dio2. Likewise, a PCB, PBDE or related compound containing solely ortho chlorines (such as PCB-54 or BDE-54), or its strongest XB interactions at the ortho position, may target Dio3 for inhibition. These preferences will be subject to other interactions within the Dio active site. Understanding the regioselectivity of these preferences may aid in the drug design to target specific Dios.

The conformational preferences of THs and halogenated aromatics will also affect Dio binding. For instance, since PBDEs have the same diphenyl ether core as THs, they may bind to Dio in a similar fashion. Xenobiotics with large halogens, such as THs and PBDEs, may be less able to adapt their most favorable conformations to the active site due to steric interactions. PCBs lack the ether linkage connecting the phenyl rings and are classified as dioxin or non-dioxin-like based on ortho-substitutions. These two conformations differ in terms of toxicity—non-dioxin-like PCBs are only toxic at higher concentrations (>1000 nM), while dioxin-like PCBs are highly toxic and mimic the structure of TCDD. Addition of halogens to ortho positions restrain the PCB to a noncoplanar conformation due to steric clashes, leading to lower flexibility around the central C-C bond [73]. Therefore, highly-ortho chlorinated species have much lower conformational flexibility, which may impact the ability of the PCB to adapt to the active site and inhibit the protein.

While these small models provide insight on the XB interactions in the active site of Dio, the simple model itself is insufficient for describing various factors that may influence overall XB favorability. Interactions with active site residues will control regioselective binding and activation of THs and the ability of inhibitors to block the active site. For example, OH-substituted inhibitors may be accommodated by the His-Arg clamp proposed for Dio3 [7]. The X···O and X···N XB interactions to ancillary halogens may increase the stability of the TH substrate in the active site. Hydroxylated PCBs and PBDEs that can interact with this clamp and form the X···Se interaction could be the most potent inhibitors. Experimental studies of Dio with PCBs, PBDEs and related xenobiotics are necessary to further explore the relationship between selectivity, inhibition, and substitution pattern.

From the modeling perspective, simulations of full proteins will be needed to understand how these factors affect the XB interaction and substrate binding. Force fields have been developed to account for XB interactions through the use of dummy atoms to represent the anisotropic density at the halogen [117,118,119,120,121]. In addition, AutoDock VinaXB has been developed to include a halogen bonding scoring function [122]. Calculating the free energies of binding (i.e., ΔG) may also be useful for predicting favorability of protein-ligand interactions. MMPBSA and MMGBSA (and their variants) and QM methods such as fragmented molecular orbital (FMO) could be used for such calculations and may aid in drug design for suitable inhibitors to target the active site of Dios [123,124,125]. Use of these computational methods to understand the underlying mechanisms and key interactions in Dios with an eye toward designing treatments for TH-related disorders is being pursued within our group.

## Figures and Tables

**Table 1:** Interaction energies and selected distances for halogen bonding (XB) complexes of TH analogues with MeSe^−^ at each unique iodine center, adapted from reference [28].

Compound	XB Position	*d*(C–I), Å; (Δ*d*(C–I), Å)	*d*(I···Se), Å	ΔE_ZPE_, kcal mol^−1^	ΔE_D→A_, kcal mol^−1^
T_4_	Inner	2.300 (+0.198)	2.917	−29.59	53.58, 4.18 ^[a]^
T_4_	Outer	2.282 (+0.169)	2.960	−29.50	45.93, 3.83 ^[a]^
T_3_	Inner	2.299 (+0.197)	2.922	−28.43	52.87, 3.96 ^[a]^
T_3_	Outer	2.256 (+0.144)	3.006	−24.84	40.25, 3.03 ^[a]^
rT_3_	Inner	2.276 (+0.174)	2.953	−25.14	48.43, 3.17 ^[a]^
rT_3_	Outer	2.291 (+0.190)	2.946	−33.07	48.98, 4.12 ^[a]^
3,3′-T_2_	Inner	2.272 (+0.169)	2.960	−23.56	46.89, 3.20 ^[a]^
3,3′-T_2_	Outer	2.265 (+0.153)	2.990	−28.46	43.06, 3.61 ^[a]^
3,5-T_2_	Inner	2.286 (+0.184)	2.942	−26.61	48.51
3′,5′-T_2_	Outer	2.286 (+0.173)	2.954	−32.02	47.48
3-T_1_	Inner	2.262 (+0.158)	2.980	−21.41	43.43
3′-T_1_	Outer	2.260 (+0.158)	3.002	−27.29	41.18

^[a]^ Donor-acceptor energies with MeSeH from reference [96].

## References

[1] Mondal S., Raja K., Schweizer U., Mugesh G. (2016). Chemistry and Biology in the Biosynthesis and Action of Thyroid Hormones. Angew. Chem. Int. Edit..

[2] Schweizer U., Steegborn C. (2015). New insights into the structure and mechanism of iodothyronine deiodinases. J. Mol. Endocrinol..

[3] Bianco A.C., Salvatore D., Gereben B., Berry M.J., Larsen P.R. (2002). Biochemistry, Cellular and Molecular Biology, and Physiological Roles of the Iodothyronine Selenodeiodinases. Endocr. Rev..

[4] Brent G.A. (2012). Mechanisms of thyroid hormone action. J. Endocrinol. Invest..

[5] Köhrle J. (2019). The Colorful Diversity of Thyroid Hormone Metabolites. Eur. Thyroid J..

[6] Parmentier M., Libert F., Maenhaut C., Lefort A., Gerard C., Perret J., Van Sande J., Dumont J., Vassart G. (1989). Molecular cloning of the thyrotropin receptor. Science.

[7] Schweizer U., Schlicker C., Braun D., Köhrle J., Steegborn C. (2014). Crystal structure of mammalian selenocysteine-dependent iodothyronine deiodinase suggests a peroxiredoxin-like catalytic mechanism. Proc. Natl. Acad. Sci. USA.

[8] Fekete C., Lechan R.M. (2007). Negative feedback regulation of hypophysiotropic thyrotropin-releasing hormone (TRH) synthesizing neurons: Role of neuronal afferents and type 2 deiodinase. Front. Neuroendocrinol..

[9] Jinhui L., Yuan C., Wenjing X. (2017). Polybrominated diphenyl ethers in articles: A review of its applications and legislation. Environ. Sci. Pollut. Res..

[10] Chen D., Hale R.C. (2010). A global review of polybrominated diphenyl ether flame retardant contamination in birds. Environ. Int..

[11] Dorman D.C., Chiu W., Hales B.F., Hauser R., Johnson K.J., Mantus E., Martel S., Robinson K.A., Rooney A.A., Rudel R. (2018). Polybrominated diphenyl ether (PBDE) neurotoxicity: A systematic review and meta-analysis of animal evidence. J. Toxicol. Environ. Health B Crit. Rev..

[12] Lorber M. (2008). Exposure of Americans to polybrominated diphenyl ethers. J. Expo. Sci. Environ. Epidemiol..

[13] Zhao S., Rogers M.J., Ding C., He J. (2018). Reductive Debromination of Polybrominated Diphenyl Ethers—Microbes, Processes and Dehalogenases. Front. Microbiol..

[14] Hites R.A. (2004). Polybrominated Diphenyl Ethers in the Environment and in People:  A Meta-Analysis of Concentrations. Environ. Sci. Technol..

[15] Gibson E.A., Siegel E.L., Eniola F., Herbstman J.B., Factor-Litvak P. (2018). Effects of Polybrominated Diphenyl Ethers on Child Cognitive, Behavioral, and Motor Development. Int. J. Environ. Res. Public Health.

[16] Bell M.R. (2014). Endocrine-disrupting actions of PCBs on brain development and social and reproductive behaviors. Curr. Opin. Pharmacol..

[17] McGovern V. (2006). PCBs Are Endocrine Disruptors: Mixture Affects Reproductive Development in Female Mice. Environ. Health Perspect..

[18] Yang O., Kim H.L., Weon J.-I., Seo Y.R. (2015). Endocrine-disrupting Chemicals: Review of Toxicological Mechanisms Using Molecular Pathway Analysis. J. Cancer Prev..

[19] Matthiessen P., Wheeler J.R., Weltje L. (2018). A review of the evidence for endocrine disrupting effects of current-use chemicals on wildlife populations. Crit. Rev. Toxicol..

[20] Vuong A.M., Braun J.M., Webster G.M., Zoeller R.T., Hoofnagle A.N., Sjödin A., Yolton K., Lanphear B.P., Chen A. (2018). Polybrominated diphenyl ether (PBDE) exposures and thyroid hormones in children at age 3 years. Environ. Int..

[21] Roberts S.C., Bianco A.C., Stapleton H.M. (2015). Disruption of Type 2 Iodothyronine Deiodinase Activity in Cultured Human Glial Cells by Polybrominated Diphenyl Ethers. Chem. Res. Toxicol..

[22] Kato Y., Ikushiro S., Haraguchi K., Yamazaki T., Ito Y., Suzuki H., Kimura R., Yamada S., Inoue T., Degawa M. (2004). A Possible Mechanism for Decrease in Serum Thyroxine Level by Polychlorinated Biphenyls in Wistar and Gunn Rats. Toxicol. Sci..

[23] Schnitzler J.G., Celis N., Klaren P.H.M., Blust R., Dirtu A.C., Covaci A., Das K. (2011). Thyroid dysfunction in sea bass (Dicentrarchus labrax): Underlying mechanisms and effects of polychlorinated biphenyls on thyroid hormone physiology and metabolism. Aquat. Toxicol..

[24] Stapleton H.M., Kelly S.M., Pei R., Letcher R.J., Gunsch C. (2009). Metabolism of Polybrominated Diphenyl Ethers (PBDEs) by Human Hepatocytes in Vitro. Environ. Health Perspect..

[25] Curtis S.W., Terrell M.L., Jacobson M.H., Cobb D.O., Jiang V.S., Neblett M.F., Gerkowicz S.A., Spencer J.B., Marder M.E., Barr D.B. (2019). Thyroid hormone levels associate with exposure to polychlorinated biphenyls and polybrominated biphenyls in adults exposed as children. Environ. Health.

[26] Domingo J.L. (2006). Polychlorinated diphenyl ethers (PCDEs): Environmental levels, toxicity and human exposure: A review of the published literature. Environ. Int..

[27] Jacobson M.H., Darrow L.A., Barr D.B., Howards P.P., Lyles R.H., Terrell M.L., Smith A.K., Conneely K.N., Marder M.E., Marcus M. (2017). Serum Polybrominated Biphenyls (PBBs) and Polychlorinated Biphenyls (PCBs) and Thyroid Function among Michigan Adults Several Decades after the 1973–1974 PBB Contamination of Livestock Feed. Environ. Health Perspect..

[28] Marsan E.S., Bayse C.A. (2017). Halogen-Bonding Interactions of Polybrominated Diphenyl Ethers and Thyroid Hormone Derivatives: A Potential Mechanism for the Inhibition of Iodothyronine Deiodinase. Chem. Eur. J..

[29] Wang X., Yang H., Hu X., Zhang X., Zhang Q., Jiang H., Shi W., Yu H. (2013). Effects of HO-/MeO-PBDEs on Androgen Receptor: In Vitro Investigation and Helix 12-Involved MD Simulation. Environ. Sci. Technol..

[30] Siddiqi M.A., Laessig R.H., Reed K.D. (2003). Polybrominated Diphenyl Ethers (PBDEs): New Pollutants–Old Diseases. Clin. Med. Res..

[31] Schecter A., Pavuk M., Päpke O., Ryan J.J., Birnbaum L., Rosen R. (2003). Polybrominated diphenyl ethers (PBDEs) in U.S. mothers’ milk. Environ. Health Perspect..

[32] Pearce E.N., Braverman L.E. (2009). Environmental pollutants and the thyroid. Best Pract. Res. Clin. Endocrinol. Metab..

[33] Pellacani C., Tagliaferri S., Caglieri A., Goldoni M., Giordano G., Mutti A., Costa L.G. (2014). Synergistic interactions between PBDEs and PCBs in human neuroblastoma cells. Environ. Toxicol..

[34] Streets S.S., Henderson S.A., Stoner A.D., Carlson D.L., Simcik M.F., Swackhamer D.L. (2006). Partitioning and Bioaccumulation of PBDEs and PCBs in Lake Michigan. Environ. Sci. Technol..

[35] Holma-Suutari A., Ruokojärvi P., Komarov A.A., Makarov D.A., Ovcharenko V.V., Panin A.N., Kiviranta H., Laaksonen S., Nieminen M., Viluksela M. (2016). Biomonitoring of selected persistent organic pollutants (PCDD/Fs, PCBs and PBDEs) in Finnish and Russian terrestrial and aquatic animal species. Environ. Sci. Eur..

[36] Fliedner A., Rüdel H., Jürling H., Müller J., Neugebauer F., Schröter-Kermani C. (2012). Levels and trends of industrial chemicals (PCBs, PFCs, PBDEs) in archived herring gull eggs from German coastal regions. Environ. Sci. Eur..

[37] Roberts S.C., Noyes P.D., Gallagher E.P., Stapleton H.M. (2011). Species-Specific Differences and Structure−Activity Relationships in the Debromination of PBDE Congeners in Three Fish Species. Environ. Sci. Technol..

[38] Yu H., Wondrousch D., Li F., Chen J., Lin H., Ji L. (2015). In Silico Investigation of the Thyroid Hormone Activity of Hydroxylated Polybrominated Diphenyl Ethers. Chem. Res. Toxicol..

[39] Qin W.-P., Li C.-H., Guo L.-H., Ren X.-M., Zhang J.-Q. (2019). Binding and activity of polybrominated diphenyl ether sulfates to thyroid hormone transport proteins and nuclear receptors. Environ. Sci.-Proc. Imp..

[40] Noyes P.D., Hinton D.E., Stapleton H.M. (2011). Accumulation and Debromination of Decabromodiphenyl Ether (BDE-209) in Juvenile Fathead Minnows (Pimephales promelas) Induces Thyroid Disruption and Liver Alterations. Toxicol. Sci..

[41] François A., Verreault J. (2018). Interaction between deca-BDE and hepatic deiodinase in a highly PBDE-exposed bird. Environ. Res..

[42] Mughal B.B., Fini J.-B., Demeneix B.A. (2018). Thyroid-disrupting chemicals and brain development: An update. Endocrin. Connect..

[43] Rossberg M., Lendle W., Pfleiderer G., Tögel A., Dreher E.-L., Langer E., Rassaerts H., Kleinschmidt P., Strack H., Cook R. (2000). Chlorinated Hydrocarbons. Ullmann’s Encyclopedia of Industrial Chemistry.

[44] Hens B., Hens L. (2018). Persistent Threats by Persistent Pollutants: Chemical Nature, Concerns and Future Policy Regarding PCBs–What Are We Heading For?. Toxics..

[45] McFarland V.A., Clarke J.U. (1989). Environmental occurrence, abundance, and potential toxicity of polychlorinated biphenyl congeners: Considerations for a congener-specific analysis. Environ. Health Perspect..

[46] Mennigen J.A., Thompson L.M., Bell M., Tellez Santos M., Gore A.C. (2018). Transgenerational effects of polychlorinated biphenyls: 1. Development and physiology across 3 generations of rats. Environ. Health.

[47] Chevrier J., Eskenazi B., Holland N., Bradman A., Barr D.B. (2008). Effects of exposure to polychlorinated biphenyls and organochlorine pesticides on thyroid function during pregnancy. Am. J. Epidemiol..

[48] Pavuk M., Schecter A.J., Akhtar F.Z., Michalek J.E. (2003). Serum 2,3,7,8-Tetrachlorodibenzo-p-dioxin (TCDD) Levels and Thyroid Function in Air Force Veterans of the Vietnam War. Ann. Epidemol..

[49] Gordon A., Surks M.I., Oppenheimer J.H. (1973). Thyroxine Stimulation of Amino Acid Incorporation Into Mitochondrial Protein: Differences Between In Vivo and In Vitro Effects. Acta Endocrinol..

[50] Lans M.C., Spiertz C., Brouwer A., Koeman J.H. (1994). Different competition of thyroxine binding to transthyretin and thyroxine-binding globulin by hydroxy-PCBs, PCDDs and PCDFs. Eur. J. Pharm. Environ..

[51] McKinney J.D., Waller C.L. (1994). Polychlorinated biphenyls as hormonally active structural analogues. Environ. Health Perspect..

[52] Cheek A.O., Kow K., Chen J., McLachlan J.A. (1999). Potential mechanisms of thyroid disruption in humans: Interaction of organochlorine compounds with thyroid receptor, transthyretin, and thyroid-binding globulin. Environ. Health Perspect..

[53] Meerts I.A.T.M., van Zanden J.J., Luijks E.A.C., van Leeuwen-Bol I., Marsh G., Jakobsson E., Bergman Å., Brouwer A. (2000). Potent Competitive Interactions of Some Brominated Flame Retardants and Related Compounds with Human Transthyretin in Vitro. Toxicol. Sci..

[54] Katarzyńska D., Hrabia A., Kowalik K., Sechman A. (2015). Comparison of the in vitro effects of TCDD, PCB 126 and PCB 153 on thyroid-restricted gene expression and thyroid hormone secretion by the chicken thyroid gland. Environ. Toxicol. Phar..

[55] Yang H., Chen H., Guo H., Li W., Tang J., Xu B., Sun M., Ding G., Jiang L., Cui D. (2015). Molecular Mechanisms of 2, 3′, 4, 4′, 5-Pentachlorobiphenyl-Induced Thyroid Dysfunction in FRTL-5 Cells. Plos ONE.

[56] Guo H., Yang H., Chen H., Li W., Tang J., Cheng P., Xie Y., Liu Y., Ding G., Cui D. (2015). Molecular mechanisms of human thyrocyte dysfunction induced by low concentrations of polychlorinated biphenyl 118 through the Akt/FoxO3a/NIS pathway. J. App. Toxicol..

[57] Soechitram S.D., Berghuis S.A., Visser T.J., Sauer P.J.J. (2017). Polychlorinated biphenyl exposure and deiodinase activity in young infants. Sci. Total Environ..

[58] Morse D.C., Wehler E.K., Wesseling W., Koeman J.H., Brouwer A. (1996). Alterations in Rat Brain Thyroid Hormone Status Following Pre- and Postnatal Exposure to Polychlorinated Biphenyls (Aroclor 1254). Toxicol. App. Pharmacol..

[59] Darras V.M. (2008). Endocrine disrupting polyhalogenated organic pollutants interfere with thyroid hormone signalling in the developing brain. Cerebellum.

[60] Butt C.M., Wang D., Stapleton H.M. (2011). Halogenated Phenolic Contaminants Inhibit the In Vitro Activity of the Thyroid-Regulating Deiodinases in Human Liver. Toxicol. Sci..

[61] Bertani R., Sgarbossa P., Venzo A., Lelj F., Amati M., Resnati G., Pilati T., Metrangolo P., Terraneo G. (2010). Halogen bonding in metal–organic–supramolecular networks. Coord. Chem. Rev..

[62] Parisini E., Metrangolo P., Pilati T., Resnati G., Terraneo G. (2011). Halogen bonding in halocarbon–protein complexes: A structural survey. Chem. Soc. Rev..

[63] Metrangolo P., Neukirch H., Pilati T., Resnati G. (2005). Halogen Bonding Based Recognition Processes:  A World Parallel to Hydrogen Bonding. Acc. Chem. Res..

[64] Scholfield M.R., Zanden C.M.V., Carter M., Ho P.S. (2013). Halogen bonding (X-bonding): A biological perspective. Prot. Sci..

[65] Mendez L., Henriquez G., Sirimulla S., Narayan M. (2017). Looking Back, Looking Forward at Halogen Bonding in Drug Discovery. Molecules.

[66] Bayse C.A. (2018). Halogen bonding from the bonding perspective with considerations for mechanisms of thyroid hormone activation and inhibition. New J. Chem..

[67] Cavallo G., Metrangolo P., Milani R., Pilati T., Priimagi A., Resnati G., Terraneo G. (2016). The Halogen Bond. Chem. Rev..

[68] Politzer P., Lane P., Concha M.C., Ma Y., Murray J.S. (2007). An overview of halogen bonding. J. Mol. Model..

[69] Costa P.J., Nunes R., Vila-Viçosa D. (2019). Halogen bonding in halocarbon-protein complexes and computational tools for rational drug design. Expert Opin. Drug Discov..

[70] Scholfield M.R., Ford M.C., Carlsson A.-C.C., Butta H., Mehl R.A., Ho P.S. (2017). Structure–Energy Relationships of Halogen Bonds in Proteins. Biochemistry.

[71] Bayse C.A., Rafferty E.R. (2010). Is Halogen Bonding the Basis for Iodothyronine Deiodinase Activity?. Inorg. Chem..

[72] Manna D., Mugesh G. (2012). Regioselective Deiodination of Thyroxine by Iodothyronine Deiodinase Mimics: An Unusual Mechanistic Pathway Involving Cooperative Chalcogen and Halogen Bonding. J. Am. Chem. Soc..

[73] Marsan E.S., Bayse C.A. (2020). Halogen Bonding Interactions of Polychlorinated Biphenyls and the Potential for Thyroid Disruption. Chem. Eur. J..

[74] Wang Y., Wang J., Li G.-X., He G., Chen G. (2017). Halogen-Bond-Promoted Photoactivation of Perfluoroalkyl Iodides: A Photochemical Protocol for Perfluoroalkylation Reactions. Org. Lett..

[75] Brammer L. (2017). Halogen bonding, chalcogen bonding, pnictogen bonding, tetrel bonding: Origins, current status and discussion. Faraday Discuss..

[76] Wang H., Wang W., Jin W.J. (2016). σ-Hole Bond vs. π-Hole Bond: A Comparison Based on Halogen Bond. Chem. Rev..

[77] Robertson C.C., Wright J.S., Carrington E.J., Perutz R.N., Hunter C.A., Brammer L. (2017). Hydrogen bonding vs. halogen bonding: The solvent decides. Chem. Sci..

[78] Chan Y.-C., Yeung Y.-Y. (2018). Halogen Bond Catalyzed Bromocarbocyclization. Angew. Chem. Int. Edit..

[79] Murray J.S., Politzer P. (2017). Molecular electrostatic potentials and noncovalent interactions. WIRES Comput. Mol. Sci..

[80] Wolters L.P., Schyman P., Pavan M.J., Jorgensen W.L., Bickelhaupt F.M., Kozuch S. (2014). The many faces of halogen bonding: A review of theoretical models and methods. WIRES Comput. Mol. Sci..

[81] Oliveira K.J., Chiamolera M.I., Giannocco G., Pazos-Moura C.C., Ortiga-Carvalho T.M. (2019). Thyroid function disruptors: From nature to chemicals. J. Mol. Endocrinol..

[82] Guo L.-C., Yu S., Wu D., Huang J., Liu T., Xiao J., Huang W., Gao Y., Li X., Zeng W. (2019). Disruption of thyroid hormone regulated proteins and gene expression by polychlorinated biphenyls, polybrominated diphenyl ethers and new flame retardants in residents of an e-waste region. Environ. Pollut..

[83] Clark T., Murray J.S., Politzer P. (2018). A perspective on quantum mechanics and chemical concepts in describing noncovalent interactions. Phys. Chem. Chem. Phys..

[84] Clark T., Hennemann M., Murray J.S., Politzer P. (2007). Halogen bonding: The σ-hole. J. Mol. Model..

[85] Hathwar V.R., Chopra D., Panini P., Guru Row T.N. (2014). Revealing the Polarizability of Organic Fluorine in the Trifluoromethyl Group: Implications in Supramolecular Chemistry. Cryst. Growth Des..

[86] Metrangolo P., Murray J.S., Pilati T., Politzer P., Resnati G., Terraneo G. (2011). Fluorine-Centered Halogen Bonding: A Factor in Recognition Phenomena and Reactivity. Cryst. Growth Des..

[87] Metrangolo P., Murray J.S., Pilati T., Politzer P., Resnati G., Terraneo G. (2011). The fluorine atom as a halogen bond donor, viz. a positive site. Cryst. Eng. Comm..

[88] Wolters L.P., Bickelhaupt F.M. (2012). Halogen Bonding versus Hydrogen Bonding: A Molecular Orbital Perspective. ChemistryOpen.

[89] Bigoli F., Deplano P., Ienco A., Mealli C., Mercuri M.L., Pellinghelli M.A., Pintus G., Saba G., Trogu E.F. (1999). Structure and Bonding of Diiodine Adducts of the Sulfur-Rich Donors 1,3-Dithiacyclohexane-2-thione (ptc) and 4,5-Ethylenedithio-1,3-dithiole-2-thione (ttb). Inorg. Chem..

[90] Manca G., Ienco A., Mealli C. (2012). Factors Controlling Asymmetrization of the Simplest Linear I3– and I42– Polyiodides with Implications for the Nature of Halogen Bonding. Cryst. Growth Des..

[91] Arman H.D., Rafferty E.R., Bayse C.A., Pennington W.T. (2012). Complementary Selenium···Iodine Halogen Bonding and Phenyl Embraces: Cocrystals of Triphenylphosphine Selenide with Organoiodides. Cryst. Growth Des..

[92] Mulliken R.S., Person W.B. (1969). Molecular compounds and their spectra. XXI. Some general considerations. J. Am. Chem. Soc..

[93] Wang C., Danovich D., Shaik S., Mo Y. (2017). A Unified Theory for the Blue- and Red-Shifting Phenomena in Hydrogen and Halogen Bonds. J. Chem. Theory Comput..

[94] Grabowski S.J. (2013). Hydrogen and halogen bonds are ruled by the same mechanisms. Phys. Chem. Chem. Phys..

[95] Cesario D., Fortino M., Marino T., Nunzi F., Russo N., Sicilia E. (2019). The role of the halogen bond in iodothyronine deiodinase: Dependence on chalcogen substitution in naphthyl-based mimetics. J. Comp. Chem..

[96] Manna D., Mugesh G. (2010). A Chemical Model for the Inner-Ring Deiodination of Thyroxine by Iodothyronine Deiodinase. Angew. Chem. Int. Edit..

[97] Pierangelo M., Giuseppe R. (2001). Halogen Bonding: A Paradigm in Supramolecular Chemistry. Chem. Eur. J..

[98] Pimentel G.C. (1951). The Bonding of Trihalide and Bifluoride Ions by the Molecular Orbital Method. J. Chem. Phys..

[99] Hach R.J., Rundle R.E. (1951). The Structure of Tetramethylammonium Pentaiodide1,1a. J. Am. Chem. Soc..

[100] Munzarová M.L., Hoffmann R. (2002). Electron-Rich Three-Center Bonding:  Role of s,p Interactions across the p-Block. J. Am. Chem. Soc..

[101] Sirimulla S., Bailey J.B., Vegesna R., Narayan M. (2013). Halogen Interactions in Protein–Ligand Complexes: Implications of Halogen Bonding for Rational Drug Design. J. Chem. Inf. Model..

[102] Riley K.E., Hobza P. (2011). Strength and Character of Halogen Bonds in Protein–Ligand Complexes. Cryst. Growth Des..

[103] Zhou P., Tian F., Zou J., Shang Z. (2010). Rediscovery of halogen bonds in protein-ligand complexes. Mini. Rev. Med. Chem..

[104] Schweizer U., Towell H., Vit A., Rodriguez-Ruiz A., Steegborn C. (2017). Structural aspects of thyroid hormone binding to proteins and competitive interactions with natural and synthetic compounds. Mol. Cell. Endocrinol..

[105] Eneqvist T., Lundberg E., Karlsson A., Huang S., Santos C.R.A., Power D.M., Sauer-Eriksson A.E. (2004). High Resolution Crystal Structures of Piscine Transthyretin Reveal Different Binding Modes for Triiodothyronine and Thyroxine. J. Biol. Chem..

[106] Zhou A., Wei Z., Read R.J., Carrell R.W. (2006). Structural mechanism for the carriage and release of thyroxine in the blood. Proc. Natl. Acad. Sci..

[107] Liu L., Baase W.A., Matthews B.W. (2009). Halogenated Benzenes Bound within a Non-polar Cavity in T4 Lysozyme Provide Examples of I⋯S and I⋯Se Halogen-bonding. J. Mol. Biol..

[108] Wilcken R., Zimmermann M.O., Lange A., Joerger A.C., Boeckler F.M. (2013). Principles and Applications of Halogen Bonding in Medicinal Chemistry and Chemical Biology. J. Med. Chem..

[109] Bianco A.C., Kim B.W. (2006). Deiodinases: Implications of the local control of thyroid hormone action. J. Clin. Invest..

[110] Sorimachi K., Cahnmann H.J. (1979). Formation and metabolism of 3′,5′-diiodothyronine and 3,5-diiodothyronine by cultured monkey hepatocarcinoma cells. Horm. Metab. Res..

[111] Butt C.M., Stapleton H.M. (2013). Inhibition of Thyroid Hormone Sulfotransferase Activity by Brominated Flame Retardants and Halogenated Phenolics. Chem. Res. Toxicol..

[112] Byrne S.C., Miller P., Seguinot-Medina S., Waghiyi V., Buck C.L., von Hippel F.A., Carpenter D.O. (2018). Associations between serum polybrominated diphenyl ethers and thyroid hormones in a cross sectional study of a remote Alaska Native population. Sci. Rep..

[113] Van den Steen E., den Steen E.V., Covaci A., Jaspers V.L.B., Dauwe T., Voorspoels S., Eens M., Pinxten R. (2007). Accumulation, tissue-specific distribution and debromination of decabromodiphenyl ether (BDE 209) in European starlings (Sturnus vulgaris). Environ. Pollut..

[114] Coimbra A.M., Reis-Henriques M.A., Darras V.M. (2005). Circulating thyroid hormone levels and iodothyronine deiodinase activities in Nile tilapia (Oreochromis niloticus) following dietary exposure to Endosulfan and Aroclor 1254. Comp. Biochem. Phys. C.

[115] Chauhan K.R., Kodavanti P.R.S., McKinney J.D. (2000). Assessing the Role of ortho-Substitution on Polychlorinated Biphenyl Binding to Transthyretin, a Thyroxine Transport Protein. Toxicol. App. Pharmacol..

[116] Peeters R.P., Visser T.J., Feingold K.R., Anawalt B., Boyce A., Chrousos G., Dungan K., Grossman A., Hershman J.M., Kaltsas G., Koch C., Kopp P. (2000). Metabolism of Thyroid Hormone. Endotext.

[117] Jorgensen W.L., Schyman P. (2012). Treatment of Halogen Bonding in the OPLS-AA Force Field; Application to Potent Anti-HIV Agents. J. Chem. Theory Comput..

[118] Kolář M., Hobza P., Bronowska A.K. (2013). Plugging the explicit σ-holes in molecular docking. Chem. Commun..

[119] Ibrahim M.A.A. (2011). Molecular mechanical study of halogen bonding in drug discovery. J. Comp. Chem..

[120] Rendine S., Pieraccini S., Forni A., Sironi M. (2011). Halogen bonding in ligand–receptor systems in the framework of classical force fields. Phys. Chem. Chem. Phys..

[121] Gutiérrez I.S., Lin F.-Y., Vanommeslaeghe K., Lemkul J.A., Armacost K.A., Brooks C.L., MacKerell A.D. (2016). Parametrization of halogen bonds in the CHARMM general force field: Improved treatment of ligand–protein interactions. Bioorg. Med. Chem..

[122] Koebel M.R., Schmadeke G., Posner R.G., Sirimulla S. (2016). AutoDock VinaXB: Implementation of XBSF, new empirical halogen bond scoring function, into AutoDock Vina. J. Cheminformatics.

[123] Kollman P.A., Massova I., Reyes C., Kuhn B., Huo S., Chong L., Lee M., Lee T., Duan Y., Wang W. (2000). Calculating Structures and Free Energies of Complex Molecules:  Combining Molecular Mechanics and Continuum Models. Acc. Chem. Res..

[124] Genheden S., Ryde U. (2011). Comparison of the Efficiency of the LIE and MM/GBSA Methods to Calculate Ligand-Binding Energies. J. Chem. Theory Comput..

[125] Kitaura K., Ikeo E., Asada T., Nakano T., Uebayasi M. (1999). Fragment molecular orbital method: An approximate computational method for large molecules. Chem. Phys. Lett..

